# Glucose inhibitor tubes in Croatian laboratories: are we doing well?

**DOI:** 10.11613/BM.2024.030901

**Published:** 2024-10-15

**Authors:** Alen Vrtarić, Nora Nikolac Gabaj, Ivana Ćelap

**Affiliations:** 1 Department of Cinical Chemistry, Sestre Milosrdnice University Hospital Center, Zagreb, Croatia; 2 CROQALM, Croatian center for external quality assessment, Zagreb, Croatia; 3 Faculty of Pharmacy and Biochemistry, University of Zagreb, Zagreb, Croatia

**Keywords:** preanalytical phase, glucose, glucose inhibitor, anticoagulants, citrate

## Abstract

**Introduction:**

Reliable and accurate measurement of blood glucose concentration is of crucial importance for making clinical decisions in diagnosis diabetes, gestational diabetes and impaired fasting glucose tolerance.

**Materials and methods:**

Survey was performed in form of questionnaire. Questionnaire was sent to all Croatian laboratories (N = 204) in electronic form using SurveyMonkey cloud-based software (SurveyMonkey, Inc., San Mateo, USA) as an extra-analytical module of the Croatian EQA (External Quality Assessment) provider Croatian center for external quality assessment (CROQALM) in June 2023.

**Results:**

In total 148 (73%) of laboratories responded to the survey. Large proportion of laboratories never use glucose inhibitor tubes for random glucose measurement (more than half) or for glucose function tests (one quarter). Only three laboratories use recommended glycolysis inhibitor citrate. Many other inhibitors are also used, even if some of them are not recommended for plasma glucose measurement. Glucose is almost never (93%) sampled on ice when glucose inhibitor tube is not available.

**Conclusions:**

Laboratories in Croatia do not follow the recommended procedures regarding glycolysis inhibitors for glucose determination.

## Graphical abstract

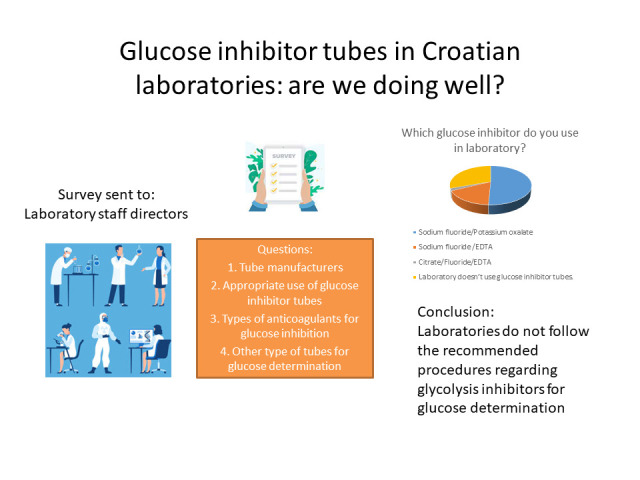

## Highlights

• Croatian laboratories do not follow the recommended procedures regarding glycolysis inhibitors for glucose measurement

• We consider that this is a potential problem affecting the results of glucose measurement

• In use are also inhibitors that are not recommended for plasma glucose measurement

## Introduction

Glucose is one of the commonly ordered analytes in clinical chemistry laboratories. Reliable and accurate measurement of blood glucose concentration is of crucial importance for making clinical decisions in diagnosis diabetes, gestational diabetes and impaired fasting glucose tolerance (*1*). Glycolysis continues *in vitro* after the blood sampling. This is the main cause of preanalytical variability for plasma glucose measurement, especially when there is some delay in analysis where sample is not centrifuged immediately after venipuncture or where the sample is not kept on ice-water slurry. Glycolysis can be prevented by adding various additives (inhibitors) to the sample tubes. Several inhibitors have been used such as sodium fluoride (NaF), sodium or lithium iodoacetate, lithium heparin tubes placed on ice slurry and blood acidification with citrate (*2*). Sodium fluoride is currently mostly used additive for inhibition of glycolysis, but it is not a very effective one. It inhibits the enzyme enolase, which is penultimate enzyme at the end of glycolytic pathway. As a result, glycolysis inhibition is delayed up to 3-4 h until all substrates for enzymes in upper glycolitic pathway are run down (*3*). Better inhibition of glycolysis can be achieved with acidification of sample with citrate to pH value of 5.3-5.9. This effect leads to immediate inhibition of enzymes involved in glycolysis at the very beginning of the pathway (hexokinase and phosphofructokinase) (*3*). Current guidelines approved by The Association for Diagnostics & Laboratory Medicine (ADLM), formerly American Association for Clinical Chemistry (AACC) and American Diabetes Association (ADA) recommended two different methods for preventing glycolysis. The first method is to collect blood into a tube containing rapidly effective glycolysis inhibitor such as granulated citrate buffer. If this procedure can not be achieved the second method is recommended. After sampling in a lithium heparin tube without glycolysis inhibitor the sample tube should be immediately placed in an ice-water slurry and the plasma separation should be completed within 30 minutes after collection. Tubes containing only enolase inhibitors, such as sodium fluoride should not be used (*4*). Second recommended procedure is less practical and not so convenient as it requires rapid sample handling after venipuncture and ice slurry. The first procedure is more convenient as it does not require fast sample handling. In response to that, a few manufacturers produced different sample tube types containing various concentrations and forms of citrate additive. Therefore, the aim of our study was to examine compliance with the new ADA recommendation among laboratories in Croatia regarding the usage of glucose inhibitors for random glucose measurement and function glucose tests (*5*, *6*).

## Materials and methods

Survey was performed in form of questionnaire. Questionnaire was sent to all Croatian laboratories (N = 204) in electronic form using SurveyMonkey cloud-based software (SurveyMonkey, Inc., San Mateo, USA) as an extra-analytical module of the Croatian EQA (External Quality Assessment) provider Croatian center for external quality assessment (CROQALM) in June 2023. Since CROQALM is mandatory for all Croatian laboratories, all kind of laboratories (private, primary care, hospital and university hospital laboratories) received a questionnaire.

## Results

In total 148 (73%) of laboratories responded and the results of the survey are presented in Table 1. Generally, a large proportion of laboratories never use glucose inhibitor tubes for random glucose measurement (more than half) or for glucose function tests (one quarter). Only three laboratories use recommended glycolysis inhibitor citrate. Many other inhibitors are also used, such as sodium fluoride/potassium oxalate, sodium fluoride/EDTA, even if some of them are not recommended for plasma glucose measurement (only EDTA alone). Glucose is almost never (93%) sampled on ice when glucose inhibitor tube is not available. The most common problem is that laboratories use different types of additives for glucose inhibition and some of them never use glucose inhibitors for functional tests (24%), which is not in compliance with recommended procedures by the ADA.

**Table 1:** Results of the survey

**Question**	**N = 148** **N (%)**
1. Please provide a name of the test tube manufacturer you are currently using in your laboratory*.	
a) Becton, Dickinson and Company (Franklin Lakes, New Jersey, USA)	41 (27.7)
b) Greiner Bio-One (Kremsmünster, Austria)	78 (52.7)
c) Sarstedt (Nümbrecht, Germany)	0 (0.0)
d) LT Burnik, Ltd (Vodice, Slovenia)	14 (9.5)
e) Kima (Arzergrande PD, Italia)	10 (6.7)
f) a) and d)	3 (2.0)
g) a), b) and d)	1 (0.7)
**2. Do you use glucose inhibitor tubes for random glucose measurement?**	
a) Yes, always.	7 (4.8)
b) Yes, sometimes.	54 (36.5)
c) No, never.	85 (57.4)
d) Glucose is not measured in laboratory.	1 (1.4)
**3. Do you use glucose inhibitor tubes for the function glucose tests (oral glucose tolerance test, fasting and postprandial glucose, HOMA index)?**	
a) Yes, always.	88 (59.5)
b) Yes, sometimes.	15 (10.1)
c) No, never.	36 (24.3)
d) Glucose function tests are not measured in laboratory.	9 (6.1)
**4. Which glucose inhibitor do you use in laboratory?**	
a) Sodium fluoride/potassium oxalate.	75 (50.7)
b) Sodium fluoride /EDTA.	25 (16.9)
c) Citrate/fluoride/EDTA.	3 (2.0)
d) Laboratory doesn’t use glucose inhibitor tubes.	45 (30.4)
**5. Other than in the glucose inhibitor tube, glucose is measured in^†^:**	
a) Serum (without separator).	59 (35.8)
b) Serum (with separator).	94 (57.0)
c) Plasma (EDTA).	3 (1.8)
d) Plasma (heparin).	5 (3.0)
e) Did not answer.	4 (2.4)
**6. When glucose is not measured in the glucose inhibitor tube, sampling on ice is done:**	
a) Yes, always.	2 (1.4)
b) Yes, sometimes.	3 (2.0)
c) No, never.	139 (93.9)
d) Glucose is always measured in the glucose inhibitor tube.	2 (1.4)
e) Did not answer.	2 (1.4)
*Answers d) to h) are generated from the free entry on the option “other”. ^†^Multiple choice question (N = 165)	

## Discussion

Cut-off values of plasma glucose concentrations are derived from studies that were performed over years and they provide evidence base for the diagnosis of diabetes. Concentration of glucose above cut-off values predict development of complications in the future. For gestational diabetes, one large study Hyperglycemia and Adverse Pregnancy Outcome (HAPO) has also defined potential risk of adverse neonatal and maternal events connected with glucose concentration (*7*). In HAPO study glucose in oral glucose tolerance test (OGTT) was measured from fluoride-oxalate tubes sampled in ice-slurry or crushed-ice, prior to plasma separation (*7*, *8*). Recommendations from Croatian Chamber of Medical Biochemists (CSMB) were adopted from HAPO study (*9*). It is important to consider how closely sample managing protocols in routine laboratory practice agree with those used in the previous studies. Protocols derived from these studies should be followed because small errors will lead to the misclassification of diabetic patients (*8*). Recommendations for cut-off values for diagnosis of diabetes were endorsed by the WHO and adopted by ADLM and ADA (*4*). Heparin plasma tubes sampled on ice were used to establish this cut-off values. Citrate tubes are not yet so available on the market. Currently, only some of the manufacturers that can offer mentioned tubes are FC mix tubes from Greiner Bio-One and S-Monovette GlucoEXACT from Sarstedt. Measurement of glucose OGTT using citrate tubes is more effective than in tubes containing NaF as inhibitor. This new citrate glycolysis inhibitor seems to be the most effective inhibitor for accurate and reliable plasma glucose measuring (*10*). The main problem with citrate containing tubes is lack of standardization and clinically significant biases are seen between different tube types used for glucose measurement (*6*, *10*). In a study performed by Saracevic *et al.*, glucose concentration that was measured in citrate inhibitor tubes is up to 7.3% higher than in ice-water slurry lithium heparin tubes. There is a clinically unacceptable bias of up to 7.1% between glycolysis inhibitor-containing tubes (*6*). Because of this difference between different glycolysis inhibitor-tubes, the use of different test tubes with glycolysis inhibitors can lead to significant and serious risk for patient safety, such as missed diagnose or misdiagnose of diabetes. Thus, it is essential to perform the re-evaluation of diagnostic criteria to ensure that patient care remains consistent despite the changes (*10*, *11*). Additionally, our survey showed that many laboratories still use NaF tubes which contrary to recommended guidelines due to their inadequate potential to immediately inhibit glycolysis. As a result, there is significant bias between NaF and citrate tubes (*5*, *11*). Lack of harmonization in glucose inhibitors can lead to confusion in interpretation and can have unwanted outcome on patient (*5*). As we can see on 6th question from our survey, sampling on ice is almost never practiced. The reasons are described above, demanding pre-analytical criteria and fast sample handling, which is almost impossible to achieve in laboratories that process a large number of samples *per* day. Glycolysis inhibitor tubes should be used for random glucose measurements, not just for OGTT and other functional tests. As we can see from our questionnaire, 85 laboratories never use glycolysis inhibitor tubes for routine (random) glucose measurement. The reason can be in reducing cost. It is more economical to use only one tube for glucose measurement and for other biochemical parameters. Despite the resulting cost, the benefit to the patient is greater to use tube with glycolysis inhibitor and should be put at the first place.

To conclude, laboratories in Croatia do not use appropriate glycolysis inhibitors for accurate glucose determination. We recommend that national societies of laboratory medicine propose their own recommendations before introducing new citrate tubes in routine practice.

## Data Availability

All data generated and analyzed in the presented study are included in this published article.
